# Revisiting CFTR Interactions: Old Partners and New Players

**DOI:** 10.3390/ijms222413196

**Published:** 2021-12-07

**Authors:** Carlos M. Farinha, Martina Gentzsch

**Affiliations:** 1 BioISI—Biosystems and Integrative Sciences Institute, Faculty of Sciences, University of Lisboa, 1749-016 Lisboa, Portugal; 2 Marsico Lung Institute and Cystic Fibrosis Research Center, School of Medicine, University of North Carolina, Chapel Hill, NC 27599, USA; 3 Department of Pediatrics, Division of Pediatric Pulmonology, School of Medicine, University of North Carolina, Chapel Hill, NC 27599, USA; 4 Department of Cell Biology and Physiology, School of Medicine, University of North Carolina, Chapel Hill, NC 27599, USA

**Keywords:** CFTR interactions, rare mutation, chaperones, processing, CFTR modulators, theratyping, proteostasis, folding, degradation, transcriptional regulation

## Abstract

Remarkable progress in CFTR research has led to the therapeutic development of modulators that rescue the basic defect in cystic fibrosis. There is continuous interest in studying CFTR molecular disease mechanisms as not all cystic fibrosis patients have a therapeutic option available. Addressing the basis of the problem by comprehensively understanding the critical molecular associations of CFTR interactions remains key. With the availability of CFTR modulators, there is interest in comprehending which interactions are critical to rescue CFTR and which are altered by modulators or CFTR mutations. Here, the current knowledge on interactions that govern CFTR folding, processing, and stability is summarized. Furthermore, we describe protein complexes and signal pathways that modulate the CFTR function. Primary epithelial cells display a spatial control of the CFTR interactions and have become a common system for preclinical and personalized medicine studies. Strikingly, the novel roles of CFTR in development and differentiation have been recently uncovered and it has been revealed that specific CFTR gene interactions also play an important role in transcriptional regulation. For a comprehensive understanding of the molecular environment of CFTR, it is important to consider CFTR mutation-dependent interactions as well as factors affecting the CFTR interactome on the cell type, tissue-specific, and transcriptional levels.

## 1. Introduction

**Figure 1:**
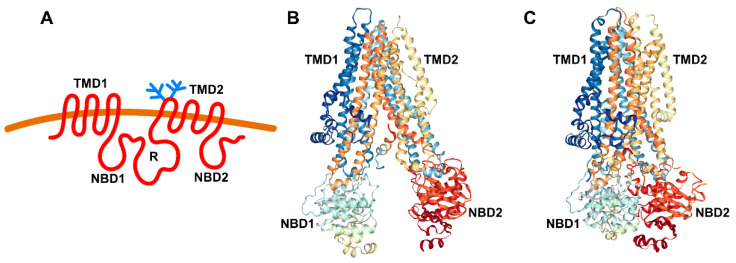
Topology and structure of CFTR. (**A**) CFTR topology. (**B**) Structure of dephosphorylated and ATP-free human CFTR (PDB ID 5UAK) [2]. (**C**) Structure of phosphorylated and ATP-bound human CFTR (PDB ID 6MSM) [3]. Images in B and C were created using NGL Viewer [4].

The CFTR gene encodes a 1480 amino acid channel protein that belongs to the ATP-binding cassette (ABC) transporter superfamily that binds ATP and promotes substrate transport across membranes. CFTR (ABCC7) contains two hydrophobic transmembrane domains (TMDs) that are each followed by a nucleotide binding domain (NBD) that resides in the cytosol (Figure 1). A special feature of CFTR is a regulatory (R) domain encoded by the central cDNA proportion of the CFTR sequence (N-terminus-TMD1-NBD1-R-TMD2-NBD1-C-terminus) (Figure 1A). The R domain has many phosphorylation sites; phosphorylation controls the conformation of CFTR (Figure 1B,C) and activity that is highly regulated.

Although the CFTR gene, which is mutated in cystic fibrosis (CF), was cloned in 1989 [1], significant progress in the development and approval of CFTR-targeting therapeutics has only occurred in the last decade. The CF research community has recently witnessed extraordinary developments with the approval of modulators to rescue the underlying defect in the most common CFTR mutation, F508del. Modulators can now be used to treat CF in up to 85–90% of individuals suffering from the disease; however, this percentage differs across the globe and is substantially lower in several countries and ethnicities. Furthermore, even the most successful modulators do not rescue the mutant CFTR function to wild-type (WT) levels. The absence of better treatments may be due to the fact that the mechanisms of action for these drugs are still poorly understood. The major problem is the significant proportion of individuals without therapeutic options available that are based on the cellular and molecular defects associated with their genotypes, which prompts a continuous interest in studying the disease mechanisms. Thus, as we have seen in the past, addressing the root of the problem is still essential. Although advances in the elucidation of CFTR structures have also been put forward recently [2,3], CFTR interactions are a puzzle that have never ceased to amaze researchers and, even with the modulators now in the equation, the problem is still unsolved. Which interactions are critical to rescue CFTR? Which ones are being altered by modulators? Which interactions are shared between common mutants and rare ones? There is ongoing interest in addressing the mechanisms through which interactions affect the CFTR biogenesis, trafficking, and function. Areas of interest to the CF research community also include studies on personalized therapies for rare CFTR mutations, insights into the regulation of CFTR expression, theratype-specific processing, and the rescue of rare mutations as well as novel interactions of CFTR that affect differentiation and development. CFTR and its function are highly affected by interactions on many levels that we aim to decipher and summarize here.

## 2. Interactions That Govern CFTR Folding, Processing, and Stability

Many membrane glycoproteins such as CFTR that are glycosylated at the endoplasmic reticulum (ER) and Golgi and finally inserted into the plasma membrane (PM) are subjected to a quality control whilst traveling through the secretory pathway [5,6,7,8,9,10]. The quality control is determined by crucial interactions either in the early secretory pathway at the ER or in the late secretory pathway at the Golgi, PM, and endosomes after CFTR has folded and exited the ER. Along the secretory pathway, many components of the proteostasis machineries interact with CFTR, regulating its folding, stabilization, or degradation and ultimately its functional protein levels [11,12,13,14,15,16,17,18].

### 2.1. Crucial Interactions in the Early Secretory Pathway

Not all CFTR proteins reach full maturation as a significant proportion of the molecule is removed by ER-associated degradation (ERAD) [10,19,20]. In this process, misfolded proteins are recognized by ER-associated molecular chaperones, then ubiquitinated, and finally transported to the proteasome for degradation. After reaching the PM, several WT-CFTRs and almost all misfolded CFTRs that are rescued from ERAD but not stabilized are subjected to endocytosis and lysosomal degradation [21,22,23]. CFTR is a multidomain protein and, therefore, the correct intramolecular interactions of TMDs and NBDs that occur co-and post-translationally during folding are crucial [24,25,26,27].

Multiple heat-shock proteins interact with CFTR during biosynthesis and are important components of its quality control. These networks are rather complex and involve a larger amount of ER and cytosolic proteins at early stages of CFTR folding. Details of these interactions have been recently reviewed [10]. Two important players that control the folding of CFTR are the ATP-binding molecular chaperones Hsc70/Hsp70 and Hsp90 [28,29,30,31,32]. When performing their function of assessing CFTR folding and scrutinizing between folded and misfolded, chaperones are assisted by co-chaperones that can help regulate the fate of CFTR. For example, the Hsc70/Hsp90 organizing protein (HOP) favors CFTR degradation by prompting interactions with destabilizing components such as CHIP, an E3 ubiquitin ligase that catalyzes CFTR ubiquitination. Although CHIP is soluble, other ERAD-related E3 ligases are membrane integrated and do not interact directly with the HSP70 chaperones. The association of CFTR with E3 ubiquitin ligase RMA1 promotes ERAD [33]. RMA1 binds to CFTR via the integral membrane protein Derlin, which may act as a retro-translocation channel and associates with multiple components of the ERAD machinery [34,35,36]. There are also folding-promoting co-factors such as HspBP1, a nucleotide exchange factor for Hsc70/Hsp70 that counteracts its interaction with CHIP and thus prevents the degradation of CFTR [37]. Hsc70/Hsp70 co-chaperones such as Hsp40 proteins (Hdj1, Hdj2, DNAJB12, cysteine string protein, etc.) can either support degradation or folding [30,33,38,39,40,41], which clearly illustrates the complex networks that fine tune the quality control of CFTR. Thus, Hsp70 and the co-chaperone networks may convert CFTR folding towards degradation whereas Hsp90 usually is an important folding-promoting chaperone [10,28,42]. However, the activation of the Hsp90 co-chaperone Aha1, a co-chaperone of Hsp90 that enhances its ATPase activity, results in the enhanced degradation of CFTR [43]. A number of small heat-shock proteins and several factors modulate CFTR biogenesis by promoting the ubiquitin or small ubiquitin-like modifier (SUMO) attachment leading to degradation by the 26S proteasome [20,28,44,45,46].

Several lectin chaperones have been shown to bind N-linked glycans of CFTR; the two best described and understood are calnexin and calreticulin. Calnexin and calreticulin bind to partially processed/trimmed core N-linked oligosaccharides. Together with the enzymes responsible for glucose removal and re-attachment, they provide an additional quality control in the ER that can modulate CFTR processing [47,48,49,50].

### 2.2. Interactions in the Late Secretory Pathway

Finally, after reaching the cell surface, CFTR is also the substrate of peripheral quality control that regulates its endocytosis in clathrin-coated vesicles. Endocytosis is facilitated by a tyrosine-based motif within the C-terminus of CFTR (YXXΦ, Φ = hydrophobic amino acid) [51] and mediated by adaptor proteins such as AP2 and Dab2 [52,53,54]. CFTR residing in endosomes is then either recycled back to the cell surface or is selected for lysosomal degradation [22,53]. Several Rab GTPases are involved in the intracellular routing of CFTR to these pathways [22,55]. These late secretory pathway interactions that occur at the cell surface concur with the peripheral protein quality control system that removes unfolded or expired CFTR from the PM, whereby the selection for lysosomal degradation is supported by the ubiquitination of CFTR [23,56].

A number of proteins containing PDZ (Postsynaptic density-95, Discs large, Zonula occludens-1 (ZO-1)) domains have been shown to bind to the C-terminal end of CFTR and affect its stability and lifetime including NHERF1, NHERF2, PDZK1, PDZK2, Shank2, and CAL [57,58,59]. Two well-studied PDZ domain proteins that enhance and decrease the half-life of CFTR are NHERF1 and CAL, respectively. NHERF1 stabilizes CFTR by linking it to apical macromolecular complexes that may also contain other regulatory proteins [60] whereas Golgi PDZ protein CAL binds to CFTR and—with the help of the SNARE protein, syntaxin 6—promotes its targeting to lysosomal degradation [61,62]. The interaction with PDZ proteins links CFTR to the ezrin (a member of the ERM (ezrin/radixin/moesin) proteins) that connects it to the actin cytoskeleton, thus orchestrating multiple interactions at the membrane. Other syntaxins and SNARE proteins were implicated in regulating CFTR trafficking and several of these components were shown to interact with the N-terminus of CFTR [63,64,65,66,67,68,69,70,71,72].

## 3. Regulation of the CFTR Function and Spatial Control of the CFTR Interactions

CFTR levels at the apical membrane are affected by its efficacy of folding and secretion, stability, and apical half-life. Several factors regulate the activity of CFTR by enhancing its apical protein levels.

### 3.1. Regulation of the Apical Levels of CFTR

Vasoactive intestinal polypeptide (VIP) has been shown to regulate CFTR secretion in the intestine [73,74,75,76] whereas cytokines affect CFTR levels in the airways; IL-1β, IL-4, TNF-α, IL-10, and IL-13 increase CFTR levels while TGF-β decreases them [77,78]. Cyclic AMP (cAMP), a well-known activator of the CFTR function (see below), also regulates CFTR levels at the cell surface through the activation of the Exchange Protein Activated by cAMP 1 (EPAC1) [79], a process that recruits several cytoskeleton regulators to the close proximity of CFTR [80].

### 3.2. Regulation of the Function of CFTR

In the airways, the activation of CFTR is regulated by airway surface liquid concentrations of adenosine and ATP to maintain fluid and liquid homeostasis, which has recently been reviewed in detail elsewhere [81]. Adenosine activates the A2B receptor leading to an intracellular increase in cAMP and the subsequent phosphorylation of CFTR whereas ATP activates the P2Y2 receptors and increases the intracellular Ca^2+^ levels that stimulate TMEM16A, a Ca^2+^-activated chloride channel; however, P2Y2 activation also stimulates CFTR.

An increasing number of kinases (e.g., Protein Kinase A, Protein Kinase C, tyrosine kinase, casein kinase) [82,83,84,85,86,87,88,89,90,91,92,93] and phosphatases (e.g., protein phosphates 1, 2A, 2B) [82,83,94,95,96,97,98,99] have been implicated in controlling the phosphorylation status of CFTR that determines its open probability. In particular, the phosphorylation of the R domain by Protein Kinase A in a cAMP-dependent manner is required by CFTR for a full function [70,86,93,100,101].

### 3.3. Spatial Control of the CFTR Interactions

In polarized airway epithelia, the spatial control of CFTR interactions adds an important regulatory element. Although cell line data contributed to the FDA approval of several CFTR modulators, it has also been demonstrated that cell lines do not always constitute a reliable physiological system for predicting effects in the primary epithelia and tissues [102,103,104,105]. This is illustrated by the effect of therapeutics that act in cell lines on premature termination mutations but not in the primary epithelia due to enhanced nonsense-mediated mRNA decay [106,107,108,109]. In differentiated cells, endogenous CFTR is spatially associated with interacting partners at specific subcellular locations at various steps of the secretory pathway as well as scaffolding proteins and regulating components including kinases, phosphatases, and components of the ubiquitination machinery [10,110,111,112,113]. Temporally and dynamically direct and indirect binding partners of CFTR that impact channel function, maturation, localization, stability, half-life, and intracellular routing differ in various instances in polarized epithelia tissues and cell lines. In addition, CFTR is not equally distributed along different locations in the airways (nasal, large vs. small airways, proximal vs. distal airways) and, furthermore, is not expressed at the same level in all cell types [114,115,116,117,118]. More recent single-cell RNA sequencing data describe the subsets of ciliated, secretory, and basal cells as well as ionocytes with various expression levels of CFTR. Although rare ionocytes express high levels of CFTR on a cellular level, in the context of complete epithelial tissues, the secretory cells appear to be the most relevant cells for mediating the CFTR function in airways [115].

## 4. Novel Roles of CFTR in Development and Differentiation

Assessing CFTR interactions has been relevant in processes such as its biogenesis, folding, trafficking, post-translational modifications, and function. Membrane stability interactions have also been shown to be crucial in understanding how CFTR contributes to the overall membrane transport homeostasis in epithelial cells, as summarized above.

### 4.1. Impact of CFTR on Development and Differentiation

Revisiting old data has recently evidenced that, apart from these obvious interactions, CFTR plays several additional functions that may derive either from its channel function or from its role as a hub for multipartner complexes, particularly at the membrane. As reviewed elsewhere [119], CFTR is known to play a role in processes such as fetal development, epithelial differentiation, polarization, and regeneration as well as being an essential player in regulating the epithelial-to-mesenchymal transition (EMT) with CF cells exhibiting a partial EMT phenotype mediated by the transcription factor TWIST1 [120]. The partial or complete absence of CFTR thus leads to an impairment of the above-mentioned processes, which may be the explanation for the elevated predisposition to cancer in CF patients. This has also led to the proposal that CFTR may function as a tumor suppressor gene [121] with evidence accumulating that a low expression of CFTR stimulates the progression of different types of cancer [122,123,124].

### 4.2. CFTR Interactions That May Support Its Novel Roles

Analyzing this information in the scope of CFTR interactions prompts the identification of those that may explain the proposed additional roles and, when absent, may account for the observed phenotypes. The observed role of CFTR in the normal differentiation of secretory cell populations in developing airways [125] has been proposed to be regulated by the interaction between CFTR and β-catenin. The absence of CFTR, and thus the lack of interaction, leads to β-catenin degradation and suppresses the activation of its signaling [126].

The role of CFTR in differentiation and polarization is probably linked to its interaction in polarized cells with an important role of the PDZ-binding domain at the C-terminus that links CFTR to the cytoskeleton. Thus, PDZ protein interactions—particularly those of NHERF1—are essential as NHERF1 is known to positively regulate actin cytoskeleton organization, thereby stabilizing CFTR at the apical membrane [127]. PDZ interactions mediate the CFTR interaction with actin [128] and thus provide a link with tight and gap junctions, once again providing a rationale for the role of CFTR in epithelial differentiation/polarization.

CFTR has also been shown to interact with the tight junction protein ZO-1. CFTR keeps (ZO-1)-associated nucleic acid binding protein (ZONAB) in tight junctions through its interaction with ZO-1, thus activating epithelial differentiation and reducing the proliferation. CFTR mutations allow ZONAB to migrate to the nucleus, leading to an increased proliferation and a decreased differentiation [129].

An interaction between CFTR and tight and adherens junction component AF-6/anadin has also been reported in colon cancer cell lines with a knockdown of CFTR leading to a reduced epithelial tightness and enhanced malignancies [130].

## 5. CFTR Gene Interactions and “Transcriptional” Regulation

When we consider interactions involving CFTR, those occurring before the protein is synthesized (i.e., interactions that regulate the CFTR gene transcription and mRNA stability) should also be acknowledged.

### 5.1. CFTR Promoter

The CFTR promoter is known to be weak and to lack elements that clearly explain its tissue specificity [131]. The CFTR promoter was described as being rich in CpG islands, containing no TATA box, having multiple transcription start sites, and containing several binding sites for the Sp1 transcription factor [132]. Sp1 may have a role in CF as it also regulates many cellular processes that are involved in/affected by CF including cell differentiation and immune responses. Interestingly, the interaction of Sp1 with the CFTR promoter is affected by the presence of promoter variants apparently leading to decreased Sp1 binding and transcriptional activity [133].

### 5.2. 3D Structure of Chromatin

One of the most relevant interactions at the gene level is that with the CCCTC-binding factor (CTCF). The primary role of CTCF is to regulate the 3D structure of chromatin, promoting the formation of loops and anchoring DNA to the nuclear lamina, thus contributing to defining the boundaries between active and heterochromatic DNA. CTCF mediates the insulator function at the CFTR locus [134], contributing to regulating the transcriptional activity. In orchestrating the CFTR locus architecture, CTCF works together with another structural complex, cohesin [135]. Cohesin has a role in stabilizing the interactions between the promoter and cis-acting intronic elements including the enhancers [135].

### 5.3. Transcription Factors

Other relevant interactions occur in specific cell types and involve the recruitment of different transcription factors to cis-regulatory elements. In airway cells, factors such as immune mediator interferon regulator factor 1 and 2 (IRF1/2), nuclear factor Y (NF-Y), or nuclear factor erythroid 2-like 2 (Nrf2) all participate in the regulation of the CFTR expression [136]. Interestingly, the overall low expression of CFTR in the lung epithelia is at least partly explained by a large variety of repressive transcription factors with Kruppel-like factor 5 (KLF5) or ets homologous factor (EHF) being among the most relevant [137]. The recent identification of pulmonary ionocytes as a rare cell type expressing high levels of CFTR in the lung also came with the identification of co-expression of the transcription factor forkhead box I1 (FOXI1) although the regulatory mechanisms underlying this expression are not yet solved [118,138]. In intestinal epithelial cells, the two major cis-regulatory elements driving the CFTR expression are recognized by factors such as hepatocyte nuclear factor 1α (HNF1α), forkhead box protein A1/As (FOXA1/A2), or caudal type homeobox 2 (CDX2), which seem to be essential for maintaining high levels of CFTR expression in these cells [139,140]. The mechanisms for CFTR expression in other organs are less understood.

### 5.4. CFTR mRNA and Interacting microRNAs

A second relevant level of interactions that control the overall CFTR expression occurs at the mRNA level. As well as obvious protein interactions regulating splicing, nuclear-to-cytoplasm transport, and translation, CFTR mRNA stability is largely regulated by microRNA (miRNA) interactions. Several miRNAs have been described as interacting with CFTR mRNA, modulating its expression levels. Changes in miRNA levels have been described in association with CF disease and are one of the possible explanations for the wide phenotypic variability observed among CF patients.

miR-101 and miR-494 were two of the first miRNAs described that interact with CFTR 3′UTR. The two miRNAs inhibit the expression of a reporter construct containing CFTR 3′UTR with a combined effect of an 80% reduction in activity [141]. These two miRNAs were later shown to be induced by cigarette smoke and further evidence suggests that a chronic cigarette smoking-induced decrease of CFTR expression in chronic obstructive pulmonary disease (COPD) patients is partially mediated by the upregulation of miR-101 [142].

The regulation of the CFTR expression through the interaction with miR-101 has also been explored in the context of novel therapeutic approaches to increase the CFTR expression. miR-101 has been successfully targeted in lung cells with a peptide nucleic acid (PNA) carrying a full complementary sequence, leading to the upregulation of the CFTR expression [143].

miR-145 is another miRNA shown to control the CFTR expression and to regulate, along with miR-101, the fetal to adult CFTR expression change, making them suitable targets for CF handling [144,145].

miR-494 and miR-509-3p act cooperatively in regulating CFTR expression. Interestingly, upon infecting non-CF airway epithelial cells with *Staphylococcus aureus* or upon stimulating them with the proinflammatory cytokines TNF-α or IL-1β, the expression of these two miRNAs was increased, leading to a concurrent decrease in the CFTR expression and function, suggesting that inflammatory mediators may regulate these miRNAs. Transfecting epithelia with anti-miRs for miR-509-3p and miR-494 or inhibiting NF-κB signaling before stimulating the cells with TNFα or IL-1β suppressed these responses, suggesting that the expression of both miRNAs was responsive to NF-κB signaling [146].

Interestingly, miR-138 has an indirect effect on CFTR expression in the opposite direction. miR-138 was shown to decrease the levels of the transcriptional repressor SIN3A, leading to an increase of CFTR mRNA and protein levels and even to rescue the F508del-CFTR function in CF primary bronchial cells [147]. This was later shown to occur through a change in the gene expression, mainly in the genes encoding chaperones, unfolded protein response mediators, and components of the ubiquitin-proteasome pathway [148].

## 6. Mutation-Specific Interactions

**Figure 2:**
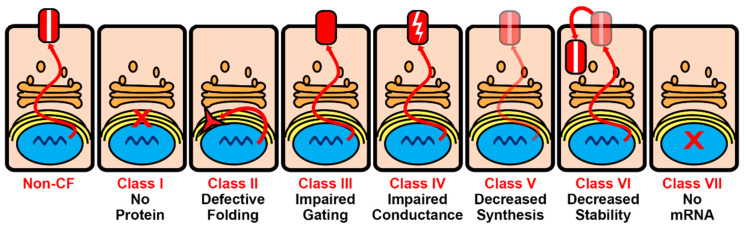
CFTR mutation classification. Non-CF: CFTR reaches the apical surface where it transports chloride and bicarbonate. Class I: nonsense mutations lead to no CFTR production. Class II: mutations impair the proper folding of CFTR and lead to ER retention and degradation. Class III: CFTR channel gating is altered, reducing CFTR open probability. Class IV: ion conductance of CFTR is diminished. Class V: reduced amounts of functional CFTR are present at the apical surface. Class VI: the apical membrane residence time of CFTR is decreased. Class VII: large deletions and other mutations lead to a lack of full-length CFTR mRNA.

Identifying the components that interact with CFTR may contribute to the specification of therapeutic targets, particularly when a focus is given to those interactors that are affected by CFTR mutations. Seven different classes of CFTR mutations have been previously described that lead to an absence of CFTR protein production (class I), an impaired proper folding and ER retention and degradation (class II), an altered CFTR regulation: reduced CFTR open probability (class III) or a diminished ion conductance (class IV), a reduced synthesis and amounts of functional CFTR present at the apical surface (class V), a decreased apical membrane residence time (class VI), and an absence of full-length CFTR mRNA production (class VII) [149,150] (Figure 2).

### 6.1. Interactome of F508del-CFTR

Early studies showed that the CFTR interaction with molecular chaperones such as Hsp70 [42], Hsp90 [28], or calnexin [151] was affected in the presence of F508del. Those initial studies were later expanded using mass spectrometry to define global protein interactions (the so-called “CFTR interactome”). Once again, the co-chaperone interactions of F508del-CFTR and WT-CFTR—particularly in the context of Hsp90 activity—were found to differ, suggesting a kinetic restriction of the mutant protein to a folding intermediate in the ER. Interestingly, a decrease in the intracellular levels of the co-chaperone Aha1, which was found to be increased in the F508del-CFTR interactome, was found to partially rescue the trafficking of the mutant protein to the PM and to restore the channel function [43]. Later observations in a more relevant cell model—bronchial epithelial cell lines vs. the HEK293 cells used in the former study—confirmed the existence of a F508del-CFTR mutation-specific interactome, mainly characterized by the gain of novel interaction partners. Protein interactions involved in the insertion of proteins into the ER (translocation), N-glycosylation, protein transport and trafficking, and anchoring at the PM as well as endocytic recycling were found to be strongly altered [152].

Similar studies characterizing the F508del-CFTR-specific interactome, either in comparison with that of WT-CFTR [153] or the mutants that escaped the ER quality control (ERQC) such as the abrogation of the retention motifs arginine-framed tripeptides [154,155], identified specific components of the signaling pathways (with a focus on the PI3K/Akt/MTOR pathway) as being increased in F508del and again with an increase in the chaperones and components of the protein degradation machineries.

### 6.2. Interaction Profiles of Rare CFTR Mutations

Recently, there has been focus not only on F508del but also on other CF-causing mutations. A study by Hutt and collaborators reported on the protein interaction profiles of CFTR bearing class II mutations G85E, F508del, R560T, and N1303K, and the class III mutation G551D. The results showed that the interactomes of CFTR with class II mutations were more closely related to one another than to either WT- or G551D-CFTR [156]. Class II mutants exhibited, as before, an increased interaction with the degradation machinery and proteostasis network components whereas G551D-CFTR, despite its ability to traffic to the PM, suffered from an overall reduction in binding affinity compared to WT-CFTR, suggesting a lack of key interactions that could explain its channel gating defect. Previously, it was shown that G551D-CFTR anchoring at the PM needed more bound actin compared with WT-CFTR [157].

The ability to rescue CFTR with different strategies has also prompted research on the effect of CFTR rescue on its interaction profile. Several of the above-mentioned studies have shown that low temperature incubation, known to rescue CFTR trafficking [158] due to an overload of the ERQC [159], or treatment with modulators such as VX-809 and VX-770 reshape the mutant protein interactome, bringing it closer to resembling the WT form [152,156]. Identifying mutation-specific protein interactions may also provide a clue as to why several mutants respond to modulators and others do not, even among those belonging to the same class. That is the case for N1303K-CFTR, the second most common class II mutation, which is more difficult to correct than F508del-CFTR. Recent data have shown that N1303K-CFTR is a client of the chaperone-co-chaperone Hsp70-DNAJB12 (being a transmembrane Hsp40/J-domain protein) following a degradation route distinct from F508del-CFTR [160]. Very recent unpublished data suggest that treatment with modulators reshapes the interactome in a way that correlates with modulation efficacy; highly responsive variants such as P67L or L206W exhibit a more WT-like interactome after a VX-809 treatment than low responding mutants such as F508del [161].

Considering CFTR interactions in the context of mutations and the responses to modulators brings to our attention the interactions with drugs. Although the binding sites for the different modulators are not completely clear, evidence has accumulated on the most probable locations. VX-809 has been suggested to bind to a TMD1 groove [162] and to a binding site at NBD1, promoting allosteric coupling to the intracellular loop 4/NBD1 interface that is directly affected by F508del [159,163,164,165]. Modulator VX-661 has been proposed to target the same CFTR interaction. There is much less data on the novel modulator VX-445, which was suggested to bind a site at NBD1 [166], which would involve a close contact with residue H620 [165]. VX-770 has been proposed to bind to two potential binding sites at the interface of the TMD1 and TMD2 of CFTR [167].

## 7. Conclusions

**Figure 3:**
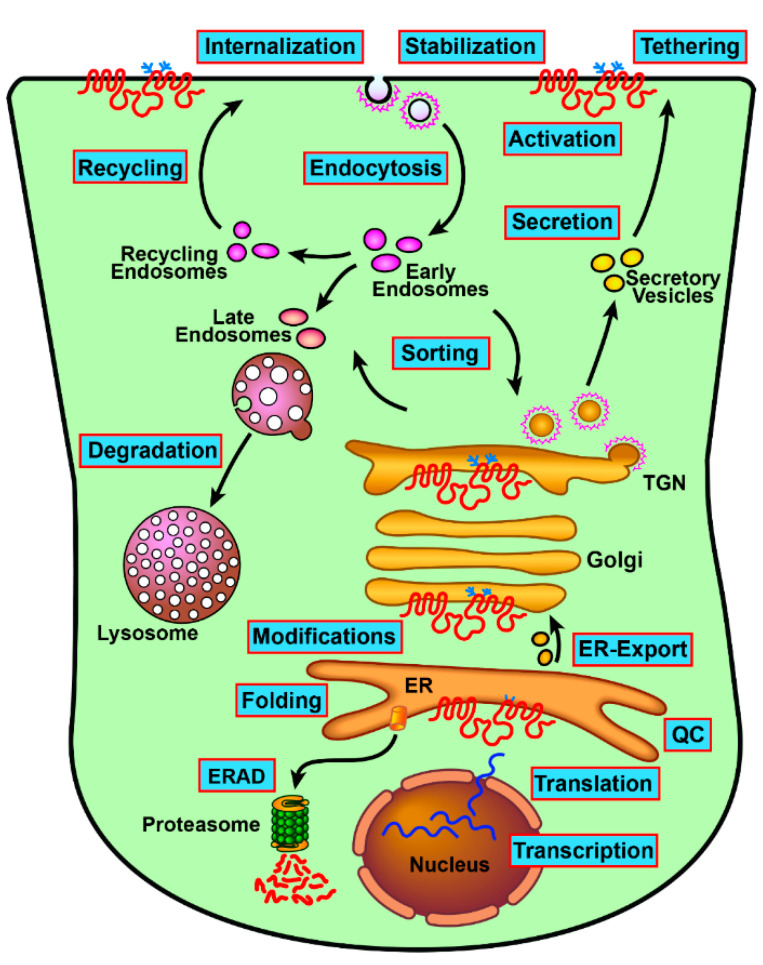
Impact of CFTR interactions throughout its lifetime. The interactome affects the fate of CFTR at multiple phases during its intracellular trafficking. Post-translational modifications include glycosylation, ubiquitination, and sumoylation. QC = quality control.

Protein–protein interactions play a critical role in the many processes that regulate CFTR proteostasis (Figure 3). CFTR folding, trafficking, stability, and functional regulation are modulated by many factors that are fine tuned to regulate the protein levels and activity. Therefore, CFTR mRNA levels may not directly correlate with the channel activity. We have learned that the CFTR interactome may be affected by many factors including patient age, gender and disease status, and that genetic modifiers may affect the disease severity [168,169,170,171,172,173,174]. Rare CFTR mutations behave dissimilarly in regard to their interactions as they may affect specific features of the CFTR molecule and its network. Although effective therapies are now available to treat most CFTR mutations, basic research on CFTR cell biology and its interactions continues to advance our knowledge about this intriguing channel whose function in development and cancer are just starting to be unraveled.
